# Optimized nonionic emulsifier for the efficient delivery of astaxanthin nanodispersions to retina: *in vivo* and *ex vivo* evaluations

**DOI:** 10.1080/10717544.2019.1682718

**Published:** 2019-11-21

**Authors:** Lei Xu, Haixiang Yu, Hongbin Sun, Xiang Yu, Ye Tao

**Affiliations:** a Department of Thoracic Surgery, China-Japan Union Hospital of Jilin University, Changchun, China;; b Department of Otorhinolaryngology, Jinling Hospital, Clinical Hospital of Medical College, Nanjing University, Nanjing, China;; c Department of Physiology, Basic Medical College, Zhengzhou University, Zhengzhou, China

**Keywords:** Medicine delivery, nano-astaxanthin, neurodegenration, therapeutics

## Abstract

Astaxanthin (AST) is a naturally occurring carotenoid with potent anti-oxidative and anti-inflammatory potency against chronic diseases. In this study, we suspended AST in different nonionic emulsifiers to produce nanodispersions. The basic physicochemical properties of the produced AST nanodispersions were verified to select the optimized nonionic emulsifier. Among the tested emulsifiers, Polysorbate 20 produced the AST nanoemulsions with smaller particle diameters, narrower size distributions, and higher AST contents among these emulsifiers. The *N*-methyl-*N*-nitrosourea (MNU) administered mouse is a chemically induced retinal degeneration (RD) model with rapid progress rate. AST suspended in Polysorbate 20 was demonstrated to ameliorate the dramatic consequences of MNU on retina architectures and function in several different tests encompassing from electrophysiology to histology and molecular tests. Furthermore, the multi-electrodes array (MEA) was used to detect the firing activities of retinal ganglion cells within the inner retinal circuits. We found that AST nanodispersions could restrain the spontaneous firing response, enhance the light induced firing response, and preserve the basic configurations of visual signal pathway in degenerative retinas. The MEA assay provided an appropriate example to evaluate the potency of pharmacological compounds on retinal plasticity. In summary, emulsifier type affects the basic physicochemical characteristic of AST nanodispersions. Polysorbate 20 acts as an optimized nonionic emulsifier for the efficient delivery of AST nanodispersions to retina. AST nanodispersions can alleviate the photoreceptor loss and rectify the abnormities in visual signal transmission.

## Introduction

Inherited retinal degeneration (RD) comprises a genetically and phenotypically heterogeneous group of retinopathies that causes night blindness, visual field constriction, visual acuity impairments in patients (Hartong et al., 2006; Souzeau et al., 2018). Thus far, the specific molecular basis underlying the RD remains unknown and no existed therapy can halt the progression of photoreceptor loss. Emerging evidences show that more than 160 types of gene mutations are implicated in the pathology of RD. This tremendous heterogeneity is problematic for any therapeutic strategies that seek to deal with the initial gene defects (Moreno et al., 2018). In the absence of specific genetic information for a given RD patient, pharmacologic therapy could be a temporizing measure until a genetic diagnosis can be found and a specific therapy devised (Drack et al., 2012). Another great hope and promise for RD patients are the stem cell-based therapy that could replace the missing retinal cells (MacLaren & Pearson, 2007). In preclinical studies, photoreceptor cell precursors and retinal pigment epithelium cells were differentiated and transplanted into animal models. However, the cellular phenomena that underlie retinal regeneration and differentiation require in-depth examination (Jeon & Oh, 2015). In RD pathogenesis, the etiologic mutations affect exclusively the rod-specific genes, but the primary rod apoptosis is often followed by the cone death, which causes severe impairments to the high acuity central vision of RD patients (Narayan et al., 2016). Typically, rods are the metabolically active consumers of oxygen supply in retina (Campochiaro & Mir, 2018). Once there is critical reduction in the number of rods, the oxygen consumption is drastically reduced, leading to a prominent elevation of oxygen tension in the distal retina layers. The resulted hyperoxia is toxic as it enhances the activity of NADPH oxidase and generates a bursts of reactive oxygen species (ROS) within degenerative retinas (Demontis et al., 2002). Photoreceptors are extremely vulnerable to ROS insults owing to the abundant polyunsaturated fatty acids in cellular membranes (Léveillard & Sahel, 2017). Moreover, oxidative damages the perturb redox metabolism in mitochondria, increase the mitochondrial membrane permeability and trigger off the cytochrome c leakage. These disturbances have been defined as pivotal initiators for the downstream caspase-mediated apoptotic cascades (Doonan et al., 2012). Pioneer researchers have endeavored to develop an optimized antioxidant formula that could provide benefit to RD patients (Grover & Samson, 2014; Tao et al., 2016). They hypothesized that the potent antioxidants might maintain suitable environment for photoreceptor survival, thereby revolutionizing the pathological process and sparing the central vision of RD patients. In the past decades, RD animal model affords an excellent platform to explore the pathological mechanisms and to screen potential therapeutic molecules. The *N*-methyl-*N*-nitrosourea (MNU) administered mice serves as a pharmacologically induced RD model with rapid progress rate (Tsubura et al., 2010). The MNU induced RD model is characterized by the extinguished electroretinogram (ERG) waveform, disorganized retinal structure and visual signal pathway remolding (Tsuruma et al., 2012). Many of these hallmarks are similar to those in human RD. The mechanism underlying MNU induced photoreceptor death is the principal alkylation of DNA, depending on the action of alkyladenine DNA glycosylase (Aag; Harrison et al., 2007). MNU toxicity could induce 7MeG and 3MeA DNA lesions, both of which are Aag substrates, and photoreceptors would die when the base excision repair machinery can no longer operate efficiently. Since MNU is a lethal toxicant with potent carcinogenic capacity, and a dramatic increase in plasmatic level would cause tumors in several organs and even animal death (Buecheler & Kleihues, 1977; Schleicher et al., 1996; Tsubura et al., 2011). Thus, the MNU is generally delivered via the intraperitoneal route to minimize the number of animals and their sufferings (Moriguchi et al., 2003). It becomes evident that the oxidative stress is incriminated in the pathological process of MNU induced RD models (Tsuruma et al., 2012; Hughbanks-Wheaton et al., 2014). Therefore, it is feasible to use substances possessing anti-oxidative actions to alleviate the photoreceptor degeneration.

Astaxanthin (AST) is a member of non-provitamin A xanthophyll carotenoids with predominantly marine origins (Ambati et al., 2014). It is widely distributed in shellfish, algae, crustaceans, and various plants. As a lipophilic molecular, AST can penetrate the capillary endothelial cell to reach these tissue that need it the most, including the central neural system, retina, and skeletal muscle (Wu et al., 2015; Feng et al., 2018). Several lines of evidences suggest that AST confers cytoprotective activities against cancers, diabetes, cardiovascular diseases, neurodegenerative disorders, and acute traumas (Liu et al., 2014; Gammone et al., 2015; Régnier et al., 2015). These favorable effects of AST are closely associated with its oxidative, anti-apoptotic, and anti-inflammatory characteristics. AST contains a unique molecular structure with conjugated double bonds on each ionone ring, giving this molecule potent anti-oxidative ability by donating electrons and terminating free radical chain reactions (Galasso et al., 2018). In particular, AST can also modulate the expressions levels of oxidation genes. An insightful study shows that AST activates the nuclear erythroid 2-related factor 2(Nrf2)-antioxidant response element signal pathway to enhance the expressions of antioxidant genes like NADPH: quinine oxidoreductase-1, heme oxygenase-1, and glutathione-S-transferase-α1 (Wu et al., 2014). AST has demonstrated potent anti-oxidative capacity in a series of pathologies, including those affecting the eye. AST can alleviate the *N*-methyl-d-aspartate-induced retinal ganglion cells (RGCs) apoptosis (Nakajima et al., 2008). AST is able to rescue the RGCs in genetically engineered diabetic animal models (Dong et al., 2013). An *in vitro* study shows that AST alleviates the H_2_O_2_-induced oxidative insults and suppresses the ROS production in retinal pigment epithelial cell cultures (Li et al., 2013). AST also scavenges the ROS and inhibits ultraviolet B-induced corneal epithelial cell apoptosis (Harada et al., 2017). In clinical practice, a dietary supplementation formula containing AST can improve the visual function of age-related macular degeneration patients (Piermarocchi et al., 2012). However, minimal data is available regarding the AST induced effects on photoreceptor apoptosis of RD. In the past decades, nanotechnology has earned a lot of research interests from the field of drug design and delivery (Mazaheri et al., 2015; Lloyd-Parry et al., 2018). Nanotechnology has been applied to enhance the bioavailability of these nature molecules with therapeutic potentials. A good understanding of the therapeutic molecular at the nanometer diameter would allow the researcher to develop drug formulation with better efficiency and safety (Voltan et al., 2016). It has been shown that the nonionic emulsifiers could be used to produce the AST nanodispersions (Anarjan & Tan 2013). The emulsifier type plays a critical role in determining the physicochemical characteristics of AST nano-dispersions. In this study, we use several types of polysorbate to produce the AST nanodispersions via an emulsification technique. The basic physicochemical properties of the produced AST nanodispersions are systemically examined. Furthermore, we evaluate the AST nanodispersions induced effects on the MNU administered mice. In order to determine the relative efficacy of the AST treatment, another well-known antioxidant, the lutein is incorporated in therapeutics trial. Lutein is reported counteract oxidative stress and inhibit the downstream pathological signals in various experimental and clinical applications (Johnson, 2014; Mares, 2016; Ajana et al., 2018). We find that lutein treatment is futile to delay the photoreceptor degeneration in the MNU administered mice. Conversely, AST treatment can alleviate the MNU induced retina degeneration effectively.

Light stimulus falling on the retina synchronously activates a large number of retinal neurons. The resulting changes in the trans-membrane voltage of local retinas can be measured with multi-electrodes array (MEA), and the recorded local field potentials (LFPs) are also interpreted as micro ERGs (Stett et al., 2003; Fujii et al., 2016; Hughes et al., 2016). MEA layouts are adapted to the special architecture of the retina. In MEA recordings, the light induced LFPs are useful to estimate the dynamics and the distribution of visual responses with respect to local retina, because their spatial decay is in the range of the inter-electrode distances, allowing almost gapless coverage of the recording area (Stett et al., 2003; Reinhard et al., 2014). MEA has been developed into a valuable tool to assess the therapeutics effects of pharmacological compounds, drug toxicity, and consequences of degeneration-related processes (Homma et al., 2009; Scelfo et al., 2012). Previous studies have demonstrated that MEA recording was more sensitive than full-field ERGs in detecting the visual impairments (Homma et al., 2009; Tao et al., 2015). Herein, the MEA recording shows profound alterations in the electrophysiological function of the MNU administered mice. AST nanodispersions can partly alter RGCs function in the MNU administered mice. These findings would advance our knowledge of AST nanodispersions, and cast light into the discovery of a novel therapy for RD.

## Material and methods

### AST nanodispersions and physicochemical analysis

AST nanodispersions were produced by the emulsification-evaporation method as described previously (Anarjan & Tan, 2013). Firstly, polysorbate emulsifiers (Fisher Scientific, Leicestershire, UK) were dissolved indeionized water under magnetic stirring to generate the aqueous phase. Subsequently, an organic phase consisting of AST (>90%, Zelang Biotech, Xian, China) was dissolved in dichloromethane and then was added to the aqueous phase at the organic:aqueous ratio of 1:9 (by weight). The mixture was then homogenized at 5000 rpm for 5 min. The produced nanodispersions were then homogenized at high-pressure (50 MPa) for two cycles. A rotary evaporator (Eyela NE-1001, Tokya Rikakikai, Tokyo, Japan) was used to remove solvent from emulsion. The organic phase diffused into the aqueous phase and formed AST nanodispersions. Furthermore, the emulsions were filtered with a membrane filter and the high performance liquid chromatography system (Agilent Technologies, Waldbron, Germany) was used to quantify the AST contents of these samples. A series dynamic light scattering particle analyzer (ZEN 1600, Malvern Instruments, Worcester, UK) was used to evaluate the mean particle diameter and the polydispersity index (PDI) of the AST nanodispersions under the guidance of the manufacturer’s instructions. A Zetasizer Nano ZS90 analyzer (Malvern Instruments, Worcester, UK) was used to determine the zeta-potential values of the AST nanodispersions.

### Animals and therapeutic design

**Figure 1.**
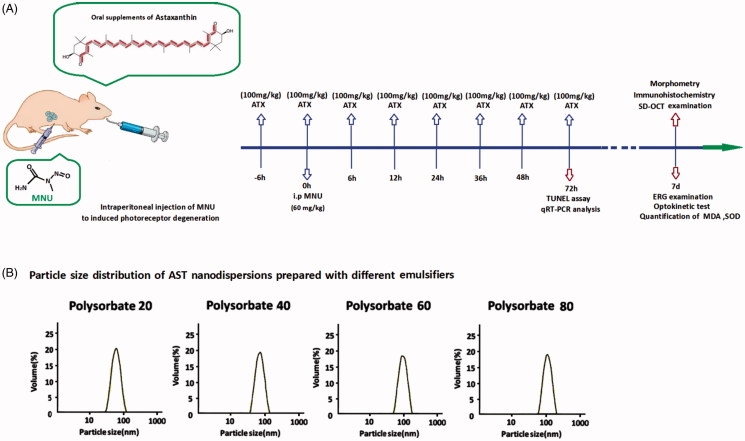
(A) A schematic illustration of the experimental protocols. Massive photoreceptor degeneration was induced by an intraperitoneal administration of MNU. AST nanodispersions were administered orally through a soft feeding tube for eight times during the therapeutic process. (B) Polysorbate 20, Polysorbate 40, Polysorbate 60, and Polysorbate 80 were used as emulsifiers to produce AST nanodispersions. These emulsifiers exhibited considerably different characteristics during homogenization. The mean particle diameters of the produced nanodispersions ranged between 70 and 160 nm, depending on the used emulsifier types. AST nanodispersions with the smallest particle diameters were produced with Polysorbate 20.

All animal procedures were conducted according to the statements of the Association for Research in Vision and Ophthalmology for the use of animals. Study protocol was reviewed and approved by the institutional animal care and use committee of the PLA General Hospital. C57BL/6 mice(Animal Center of PLA General Hospital, Beijing, China), 8-week-old with both sexes, were maintained in the air-conditioned facility (room temperature: 18 °C to 23 °C, humidity: 40% to 60%, under 12/12 h light/dark cycle; standard chow and water ad libitum).Experimental animals were divided randomly into four groups: (1) Normal control group: these mice were left without any pharmacological administration; (2) MNU group: these mice received an intraperitoneal administration of MNU(60 mg/kg; Sigma-Aldrich, MO, USA); (3) MNU + AST group: these mice received supplements of AST nanodispersions (100 mg/kg) before and after MNU administration; (4) MNU + lutein group: mice received supplements of lutein (100 mg/kg; Huile, Guangzhou, China) before and after MNU administration. The AST or lutein was administered orally through a soft feeding tube for eight times respectively at 6 h before and at 0, 6, 12, 24, 36, 48, and 72 h after MNU administration. Figure 1(A) is a schematic illustration of the experimental protocols.

### Optokinetic behavioral test

Optokinetic behavioral test was performed using a two-alternative forced choice paradigm. Mice were placed on a platform of the test machine (OptoMotry CerebralMechanics, Lethbridge, AB, Canada). Generally, each cycle of examination lasts for 3 to 5 min. Virtual cylinders were projected on the wall of the box and they turned either in clockwise or counter-clockwise direction for the mouse to track. The stepwise functions for correct track responses were used to determine the response thresholds. The initial stimulus in visual acuity measurements was set as 0.200 cyc/deg sinusoidal pattern with a fixed 100% contrast. The initial pattern in contrast sensitivity measurements was set as 100% contrast, at the fixed spatial frequency of 0.128 cyc/deg. All patterns were presented at a speed of 12 degrees/s with the mean luminance of 70 cd/m^2^.

### ERG examination

Animals were kept in dark environment for 12 h before recording. After being anesthetized with an intra-peritoneal injection of ketamine (80 mg/kg) and chlorpromazine (15 mg/kg, Shengda Animal Pharmaceutical, Jilin, China), the mice were transferred to a heating pad. Their pupils were dilated with 1% atropine and 2.5% phenylephrine hydrochloride eye drops (Xing Qi, Shenyang, China). The RETIport system (Roland Consult, Germany) with custom-made chloride silver electrodes were used for recording. Flash responses were recorded through corneal electrodes, with the reference electrode were placed in the shaven skin of the cheek, and a ground electrode clipped to the tail. White flashes with an intensity of 0.5 log cd s/m^2^ were applied to stimulate the scotopic ERGs. The inter-stimulus interval was 30 s for scotopic ERGs recording. Subsequently, the mice were light adapted for 10 min at the background intensity of 30 cd s/m^2^. Photopic ERGs were recorded at stimulus intensity of 1.48 log cd s/m^2^. The inter-stimulus interval was 0.4 s for photopic ERGs recording. Responses were filtered with high- and low-pass filters at 100 and 300 Hz, respectively. Totally 60 photopic responses and 10 scotopic were collected and averaged for analysis. Amplitude of a-wave was measured as the distance from the baseline to a-wave trough, while amplitude of b-wave was defined as the distance between trough and peak of each waveform.

### Spectral-domain optical coherence tomography

Anesthetized animals were transferred to the recording plane of an ultrahigh-resolution instrument (Bioptigen, Durham, NC, USA). Methylcellulose lubricant (Allergan Inc, Dublin, Ireland) was applied on the corneas, and the probe was positioned near the cornea until the retinal image appeared on the screen. A corresponding box was then focused on the optic nerve head (ONH) for orientation and eight measurements at the same distance (0.3 mm) from the edge of the ONH on either side were executed. Three hundred linear B-scans were obtained, and 30 averaged images were captured to achieve a better resolution. The spectral-domain optical coherence tomography cross-sectional images were analyzed subsequently with the InVivoVueTM DIVER 2.4 software (Bioptigen, Durham, NC, USA).

### MEA recording

Mice were sacrificed under dim red light. The neural retina was gently removed from eyecup and transferred into the recording chamber with the RGCs layer facing the MEA biochip. The ONH of retinal patch was placed to the middle of electrode array. Electrode array included 64 electrodes, which arranged in 8 × 8 layout with 100 μm for space configuration (Alphamed Sciences, Osaka Japan). The retina specimen was perfused with oxygenated Ringer’s solution (containing the following [in mM]: 124 NaCl, 5 KCl, 25 NaHCO_3_, 2.5 CaCl_2_, 1.15 MgSO_4_, 1.15 KH2PO_4_, and 10 d-glucose) at a flow rate of 1 mL/min. Before recording, the retinal specimen was immersed in the solution for 1 h to make them adapted to liquid environment. The MED-64 system (Alpha med Sciences, Osaka, Japan) was used to detect the analog extracellular response. The harvested neuronal signals were AC amplified, and then stored in a compatible computer for subsequent off-line software analysis. The light responses of retina specimens were elicited by a white light–emitting diode. The generated white light (at a mean photonic intensity of 850 mcd·s/m^2^) was projected onto the retinal surface via a lens focus system. For spike detection, the field potentials were wiped off through a band-pass filter (100–3000 Hz). These candidate spike waveforms were then sorted (Neuroexplorer, Nex Technologies, MA, USA). The threshold for spike detection was set to four times the standard deviation (SD) of the mean value of the measured signal for each electrode. Spike clusters were first identified using a K-mean cluster algorithm, and then manually edited for clustering errors. The signals from each channel were separated into individual units representing the spike responses from individual RGCs. Peristimulus time histograms (PSTHs) were smoothed using a Gaussian kernel to analyze the ON and OFF responses. RGCs were categorized by the PSTHs and raster plots of each unit. The response time following a light transition (onset and offset) was analyzed separately in the subpopulation of RGCs in which these response components were clearly separable. Transient ON RGC was defined as at least one spike from 50 to 300 ms after light onset in at least one-half of the light presentations. Transient OFF RGC was defined as at least one spike from 50 to 500 ms after light offset in at least one-half of the light presentations. Sustained RGC was defined as the spike rate count between 300 and 700 ms after the stimulus transition was larger than 0.2 times the count of the initial 200 ms of the response. Delayed OFF RGC had at least three spikes between the offset of light stimulus and 200 ms post offset and had a higher peak in the interval between 200 and 500 ms. The response dominance index (RDI) was calculated from the peak spike rates during the first quarter of the ON and OFF portions of the full field stimulus following a previous described method (Tian & Copenhagen, 2003; Cantrell et al., 2010). The value of the RDI ranges from –1 to 1. Cells with an RDI near 1 possess an ON response, those with an RDI near-1 possess an OFF response, and the cells with an intermediate RDI near 0 possess a more balanced ON-OFF response. Light-evoked RGCs responses were quantified as the total number of spikes occurring within 1 s following a light transition (onset or offset), after subtracting the background spontaneous firing rate over the 1 s before the stimulus. These light-evoked responses were averaged over 10 trials, and thus, the response intensity was expressed as a net mean firing rate (spikes/s).

### Histological and immunohistochemical analysis

Mice were sacrificed and their eyes were quickly removed for fixation in buffered 4% paraformaldehyde (Dulbecco’s PBS; Mediatech, Inc., Herndon, VA, USA) for 12 h. The retina was prepared under a microscope (Olympus BX 52, Tokyo, Japan) and then embedded in paraffin wax. Six retinal sections (thickness, 5 µm) cut from the ONH were processed with hematoxylin and eosin staining and were evaluated under microscope. Retinal thickness analysis was performed at 250 µm intervals along the vertically superior-inferior axis using the Image-Pro Plus software (Media Cybernetics, Silver Spring, MD, USA) Three sections were randomly selected and used for morphometric analysis. Averaged ONL thicknesses of each animal group were calculated by data from 10 eyes.

### Terminal deoxyuridine triphosphate nick-end labeling assay

Terminal deoxyuridine triphosphate nick-end labeling (TUNEL) assay was performed to detect the apoptotic cells in retinal sections following a previous described method (Yoshizawa et al., 2000). An *in situ* cell death detection POD Kit (Roche, Mannheim Germany) was used according to manufacturer’s protocol. TUNEL sections were counterstained with DAPI, mounted on slides, and then visualized with confocal microscopy (LSM510, Zeiss, Oberkochen, Germany). Apoptotic index (AI) of the ONL was calculated on the basis of cell numbers (number of TUNEL-positive nuclei/total number of photoreceptor cell nucleix100).

### Quantitative reverse transcription-polymerase chain reaction

Total RNA was extracted from retinal tissues with TRIzol reagent (Invitrogen, Gaithersburg, MD, USA) and cDNA was synthesized using μMACS™ DNA Synthesis kit (Miltenyi Biotech GmbH, Bergisch-Gladbach, Germany). All quantitative polymerase chain reaction (PCR) reactions were performed via a real-time CFX96 Touch PCR detection system (Bio-RadLaboratories, Reinach, Switzerland). The primers used in quantitative reverse transcription-PCR were: Bax: 5′-AGCTCTGAACAGATCATGAAGACA-3′ (forward) and 5′-CTCCATGTTGTTGTCCAGTTCATC3′ (reverse); Bcl-2: 5′-GGACAACATCGCTCTGTGGATGA-3′ (forward) and 5′-CAGAGACAGCCAGGAGAAATCAA3′-(reverse); Caspase-3: 5′-TGTCGATGCAGCTAACC-3′ (forward) and 5′-GGCCTCCACTGGTATCTTCTG-3′ (reverse); Calpain-2: 5′-CCCCAGTTCATTATTGGAGG-3′ (forward) and 5′-GCCAGGATTT CCTCATTCAA-3′ (reverse). The relative expression levels were normalized and quantified to obtain the ΔΔCT values (DATA assist Software v2.2, Applied Biosystems).

### Determination of antioxidant and oxidative marker levels

Superoxide dismutase (SOD) activity and malondialdehyde (MDA) concentration were measured as described previously (Du et al., 2018). The SOD activity was analyzer with the SOD Assay Kit-WST (Jiancheng Biotech Ltd., Nanjing, China). One unit(U) of SOD activity was defined as the amount of enzyme causing half inhibition in the nitroblue tetrazolium reduction rate. SOD activity was expressed as units/mg protein. The concentration of MDA was assessed using a total bile acids colorimetric assay under the guidance of the manufacturer’s protocol (Jiancheng Biotech Ltd., Nanjing, China). The manganese (Mn)-SOD activity was measured using commercially available kits under the guidance of the manufacturer’s instructions (Beyotime biotechnology, Shanghai, China). Mn-SOD activity was expressed as units/mg protein. 8-OHdG concentration was analyzed by competitive ELISA assay (R&D Systems, Minneapolis, MN, USA) according to the manufacturer’s instructions. 8-OHdG concentration was expressed asμg/mg DNA.

### Statistical analysis

Statistical difference between experimental animal groups was processed using the ANOVA analysis followed by Bonferroni’s post hoc analysis. All the *p* values are presented as mean ± SD. *p* Value < .05 was considered significant.

## Results

### Production of AST nanodispersions

As emulsifiers, the Polysorbate 20, Polysorbate 40, Polysorbate 60, and Polysorbate 80 were used to produce AST nanodispersions via emulsification technique (Figure 1(B)). The basic physicochemical properties of the produced AST nanodispersions were examined. The mean particle diameters of the produced nanodispersions ranged from 70 to 160 nm, depending on the emulsifier types (Table 1). AST nanodispersions with the smallest particle diameter were produced with Polysorbate 20. Meanwhile, the PDI value of the AST nanodispersions produced with Polysorbate 20 was significantly smaller than these produced with Polysorbate 40 or Polysorbate 60(*p* < .01; *n* = 10). Additionally, the zeta potential of the Polysorbate 20 produced AST nanodispersions was significantly smaller compared with the other three groups, suggesting that they had a more stable net surface charge (*p* < .01; *n* = 10). Table 1 also shows the AST contents of these freshly prepared nanodispersions. The AST concentration of the Polysorbate 20 produced nanodispersions was significantly higher compared with other three groups (*p* < .05; *n* = 10). Taken together, these findings suggested that the emulsifier type could affect the basic physicochemical characteristic of the resulted AST nanodispersions. Among these emulsifiers, Polysorbate 20 produced the AST nanoemulsion with smaller particle diameters, narrower size distributions, and higher AST contents. Therefore, the AST nanodispersions produced with Polysorbate 20 were used for the following therapeutic trial.

**Table 1.** The basic characteristic of AST-nanodispersions prepared with different emulsifiers.

Emulsifier	Particle size (nm)	PDI	Zeta Potential (mV)	AST concentration (mg/L)
Polysorbate 20	76.0 ± 4.1^‡§∳^	0.446 ± 0.061^‡∳^	−16.5 ± 1.9^‡§∳^	609.2 ± 4.3^‡§∳^
Polysorbate 40	85.8 ± 5.3^†§∳^	0.658 ± 0.059^†§^	−21.8 ± 2.8^†^	605.1 ± 4.0^†^
Polysorbate 60	106.6 ± 9.5^†‡∳^	0.461 ± 0.032^‡∳^	−23.9 ± 3.6^†^	601.8 ± 4.9^†^
Polysorbate 80	137.9 ± 11.6^†‡§^	0.553 ± 0.051^†§^	−22.6 ± 3.3^†^	602.1 ± 5.5^†^

^†^*p* < 0.05 for difference compared with Polysorbate 20 group.

^‡^*p* < 0.05 for difference compared with Polysorbate 40 group.

^§^*p* < 0.05 for difference compared with Polysorbate 60 group.

^∳^*p* < 0.05 for difference compared with Polysorbate 80 group.

All values are presented as mean ± SD; n = 10 per group.

### AST nanodispersions preserved photoreceptor responsiveness in degenerative retina

**Figure 2.**
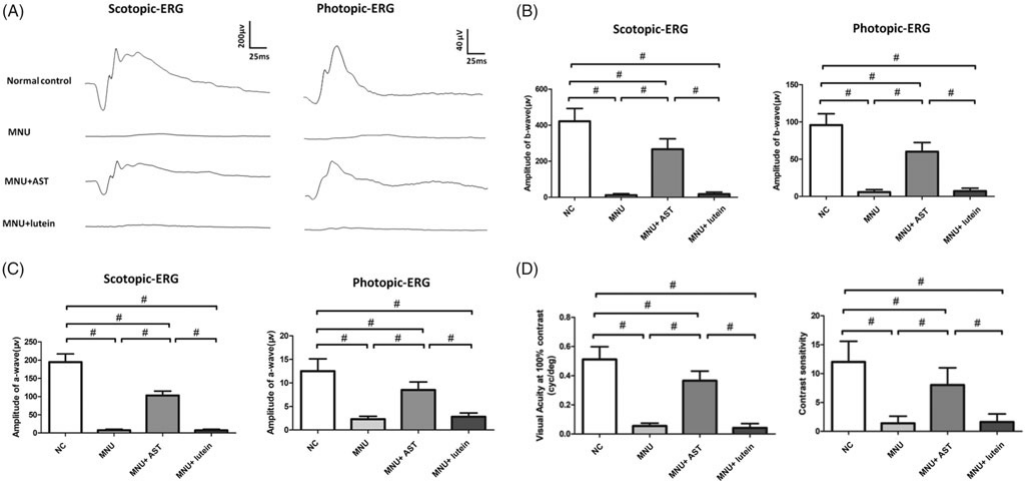
(A) Representative ERG waveforms of animal groups. AST nanodispersions produced with Polysorbate 20 were used for the following therapeutic trial. The visual function of MNU group was severely devastated. There was also a prominent reduction in the ERG responsiveness of the MNU + lutein group. By contrast, the ERG responsiveness in the MNU + AST group was less deteriorated. (B, C) The a- and b-wave amplitudes were significantly smaller in the MNU + lutein group than those in the normal controls. The a- and b-wave amplitudes were significantly greater in the MNU + AST group than those in the MNU + lutein group. (D) The visual acuity and contrast sensitivity in the MNU group were significantly smaller compared with the normal controls. The mice in the MNU + lutein group showed no significant improvement in optokinetic tests. Conversely, the visual acuity and contrast sensitivity were significantly larger in the MNU + AST group than those in the MNU group (ANOVA analysis followed by Bonferroni’s post hoc analysis was performed, ^#^*p* < .01, for differences between groups; *n* = 10).

To evaluate the AST induced effects on the visual function of MNU administered mice, scotopic and photopic ERG responses were recorded in experimental animals. The antioxidant capacity of AST is reported to be 10 times stronger than the carotenoids such as lutein, canthaxanthin, and β-carotene (Miki, 1991). Lutein is accessible to retina because of their ability to cross the blood retinal barrier. It can quench singlet oxygen and scavenge free radicals, thereby protecting the retinal neurons (Naguib, 2000). Herein, we compared the therapeutic efficacy between lutein and AST nanodispersions. The representative ERG waveforms are shown in Figure 2(A). The ERG waveforms of MNU administered group was severely devastated, as these mice exhibited no measurable scotopic or photopic ERG waveforms. There was also a prominent reduction in the b-wave amplitudes of the MNU + lutein group. The scotopic and photopic b-wave amplitudes were significantly smaller in the MNU + lutein group than those in the normal controls (scotopic: 10.8 ± 10.6 vs. 421.8 ± 71.3 µV, *p* < .01; photopic: 6.7 ± 3.9 vs. 95.8 ± 15.2 µV, *p* < .01; *n* = 10; Figure 2(B)). By contrast, the ERG waveforms in the MNU + AST group were less deteriorated (scotopic: 236.7 ± 58.0 µV; photopic: 60.1 ± 12.3 µV; *n* = 10). The scotopic and photopic b-wave amplitudes were significantly greater in the MNU + AST group than those in the MNU + lutein group (*p* < .01; *n* = 10). Comparison analysis showed that the scotopic and photopic b-wave amplitudes in the MNU + AST group were respectively ∼56.0% and ∼62.7% of the normal controls. The scotopic and photopic a-wave amplitudes were significantly larger in the MNU + AST group than those in the MNU group (scotopic: 102.9 ± 12.1 vs. 7.5 ± 3.1 µV, *p* < .01; photopic: 8.3 ± 1.7 vs. 2.8 ± 0.8 µV, *p* < .01; *n* = 10). However, the scotopic and photopic a-wave amplitudes in the MNU + lutein group were not significantly different from those in the MNU group (*p* > .05; *n* = 10). These data suggested that AST treatment could partially ameliorated the ERG impairments in MNU administered mice.

In order to determine whether the improved electrophysiological responsiveness could lead to vision improvements in a behavioral sense, we tested the experimental animals with the vision-guided optokinetic tests. The mice in the MNU group were insensitive to raster stimulus. The visual acuity and contrast sensitivity in the MNU group were both significantly smaller compared with the normal controls (*p* < .01.*n* = 10; Figure 2(C)). The mice in the MNU + lutein group showed no significant improvement in optokinetic tests. Their visual acuity and contrast sensitivity were not significantly different from those in the MNU control (*p* > .05; *n* = 10). Conversely, the visual acuity and contrast sensitivity were both significantly larger in the MNU + AST group than those in the MNU group (*p* < .01; *n* = 10), suggesting that AST nanodispersions conferred beneficial effects on the optokinetic performance of the MNU administered mice.

### AST nanodispersions inhibited photoreceptor loss in degenerative retina

**Figure 3.**
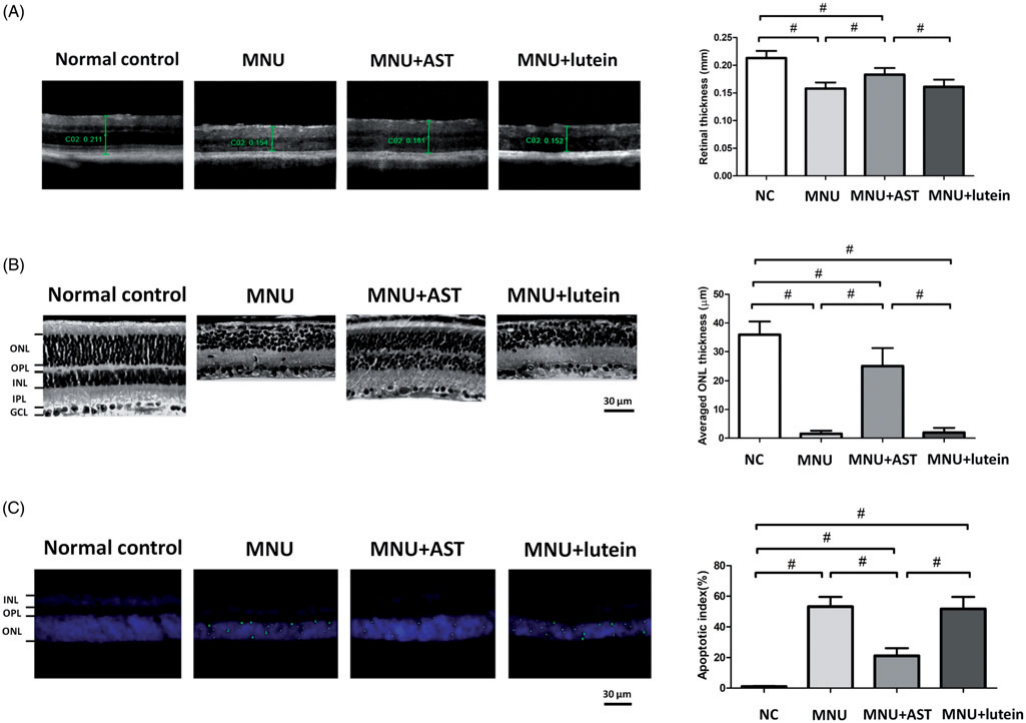
(A) OCT examination showed marked differences of the retinal thickness among the four animal groups. The retinal thickness in MNU group was not significant different from that in the MNU + lutein group. The retinal thickness in AST-treated mice was significantly larger compared with the MNU + lutein group. (B) The retina structure of the normal control group was highly organized, whereas the retinal structure of MNU group was severely destroyed. The ONL in the AST-treated mice was efficiently preserved. The average ONL thickness in MNU + AST group mice was significantly greater compared to MNU + lutein group. (C) Numerous TUNEL-labeled cells located in the ONL of the MNU group. The TUNEL-labeled cells in the MNU + AST group were prominently less compared with the MNU group. The AI of the MNU + AST group was significantly smaller compared with MNU group (ANOVA analysis followed by Bonferroni’s post hoc analysis was performed, ^#^*p* < .01, for differences between groups; *n* = 10).

In order to determine the AST induced effects on the retinal structures, OCT examination was performed *in vivo*. Differences in retinal thickness are clearly visible in the representative OCT imagines from each animal group (Figure 3(A)). The mice in the normal control group maintained thicker retinas compared to the MNU group, in which the ONL was indiscernible. The retinal thickness averaged 0.153 ± 0.011 mm in MNU group versus 0.158 ± 0.013 mm in the MNU + lutein group, and the difference was not statically significant (*p* > .05; *n* = 10).On the other hand, the mice in the MNU + AST administered group had relatively intact retinal architectures. The average retinal thickness in AST-treated mice was 0.183 ± 0.012 mm, which was significantly larger compared with the MNU + lutein group (*p* < .01; *n* = 10). The average retinal thickness in AST-treated mice was also significantly larger compared with the MNU group (*p* < .01; *n* = 10), suggesting that the AST nanodispersions was effective to preserve the retinal structure in MNU administered mice. On closer inspection, the retinal sections were visualized microscopically. The retinas of the normal control group were highly organized, whereas the retinal structure of MNU group was severely destroyed (Figure 3(B)). As expected, the ONL was not discernible in the retina sections of MNU group, indicating the complete loss of photoreceptors. On the other hand, the ONL in the AST-treated mice was efficiently preserved with relatively intact inner segment/outer segments. The average ONL thickness in MNU + AST group mice was 24.98 ± 6.27 mm compared to 1.95 ± 1.62 mm for MNU + lutein group (*p* < .01; *n* = 10). The mice of the MNU + AST group maintained a substantial proportion (∼69.5%) of ONL compared with the normal controls.

To quantify the apoptotic status of photoreceptors, TUNEL labeling was performed in retinal sections. Nuclei labeled by the TUNEL staining were rarely encountered in the retinas of normal controls (Figure 3(C)). However, numerous TUNEL-labeled cells located in the ONL of the MNU group, suggesting that MNU toxicity induced pervasive photoreceptor apoptosis in mice retinas. The TUNEL-labeled cells in the MNU + AST group were prominently less compared with the MNU group. By contrast, massive TUNEL-labeled cells were found in the ONL of the MNU + lutein group. Comparison analysis suggested that the AI was significantly larger in the MNU group than that in the normal control group (*p* < .01; *n* = 10). The AI in the MNU + lutein group was not significantly different from that in the MNU group (*p* > .05; *n* = 10). On the other hand, the AI of the MNU + AST group was significantly smaller compared with MNU group (*p* < .01; *n* = 10). These data suggested that the AST nanodispersions could inhibit the photoreceptor apoptosis in MNU administered mice.

### AST nanodispersions induced effects on the LFPs of degenerative retina

**Figure 4.**
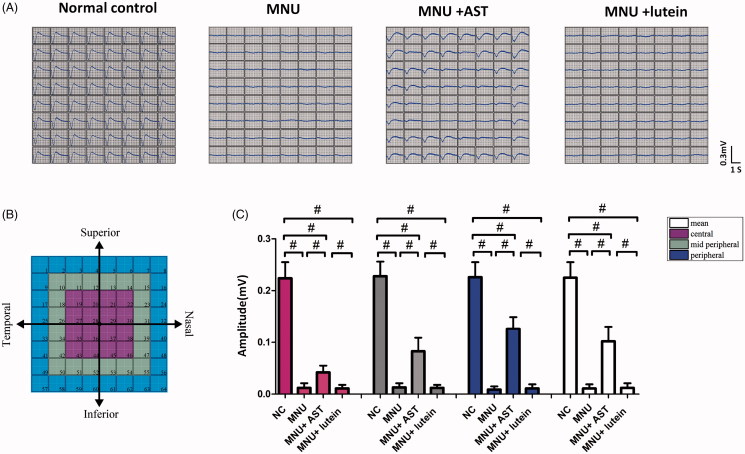
(A) Representative LFPs waveforms of each animal group. (B) Electrodes of MEA were classified into three categories: the central, the mid-peripheral, and the peripheral channels. The LFPs waveforms of the MNU group were undetectable. Conversely, the LFPs waveforms of the MNU + AST group were effectively preserved. (C) The mean amplitude of LFPs in the MNU group was significantly smaller compared with normal controls. The mean amplitude of LFPs in the MNU + AST group was significantly larger compared with the MNU group. In the MNU + AST group, the LFPs in peripheral region had larger amplitudes than those in the mid-peripheral and central regions (ANOVA analysis followed by Bonferroni’s post hoc analysis was performed, ^#^*p* < .01, for differences between groups; *n* = 10).

MNU-induced visual impairments were detected by the ERG examination. However, the ERGs represented the summed electrophysiological activities of integral retinal neurons, and the functional information on focal retina cannot be obtained (Homma et al., 2009). On closer inspection, the MEA system was used to detect the LFPs of photoreceptors. These spatial data would be valuable to assess the local functional recovery in degenerative retina. In MEA system, the electrodes were classified into three categories according to their distances to ONH: the central, the mid-peripheral, and the peripheral channels (Figure 4(B)). Typical LFPs waveforms were successfully elicited by the white light stimulus and recorded by MEA electrodes (Figure 4(A)). The main negative waveforms after the light onset represented the electrophysiological activity of photoreceptors (Tao et al., 2013). The light induced LFPs waveforms were undetectable in the mice of MNU group. On the other hand, the light induced LFPs waveforms can still be recorded in the mice of MNU + AST group, although with significantly reduced amplitude compared with normal controls (*p* < .01; *n* = 10; Figure 4(C)). In the normal control group, the waveforms of LFPs were stable during recording, and there was no positional difference among the central (0.224 ± 0.031 mV, *n* = 10) mid-peripheral (0.228 ± 0.028 mV, *n* = 10), and peripheral channels (0.226 ± 0.029 mV, *n* = 10, *p* > .05). Conversely, the LFPs waveforms were not homogeneously equal in the MNU + AST group: the mean LFPs amplitude of peripheral channels was significantly larger compared with the mid-peripheral channels (0.126 ± 0.023 mV vs. 0.083 ± 0.026 mV; *p* < .01; *n* = 10); Additionally, the mean LFPs amplitude of the mid-peripheral region was significantly larger compared with the central region (0.083 ± 0.026 mV vs. 0.042 ± 0.013 mV; *p* < .01; *n* = 10).

### AST nanodispersions induced effects on the visual signal circuits

**Figure 5.**
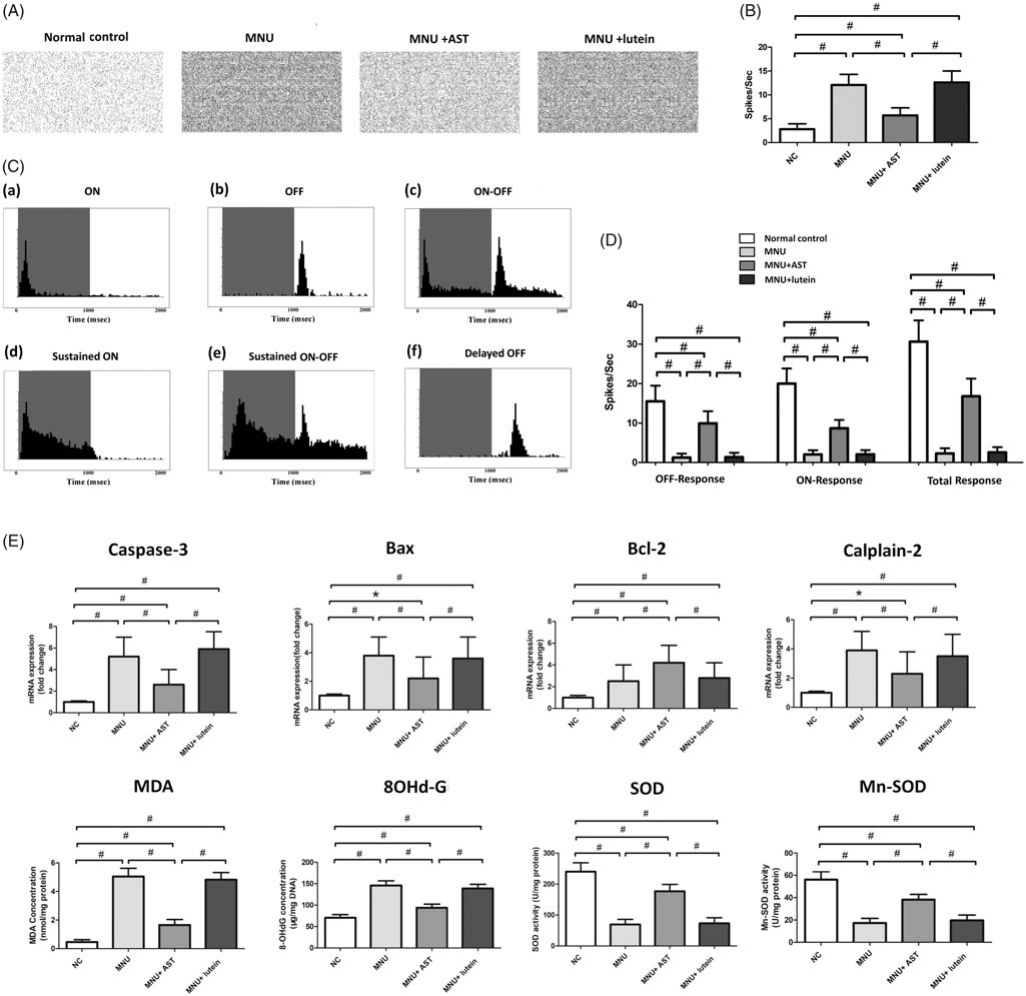
(A) Spontaneous firing spikes of RGCs were detected by MEA system. (B) The spontaneous firing rate in the MNU was significantly higher compared with the normal controls. The spontaneous firing rate in the MNU + AST group was significantly lower compared with the MNU group. (C) Six categories of RGCs were isolated according to their PSTHs. (D) The total firing rate decreased significantly in the MNU group than that in the normal controls. The total firing rate was significantly higher in the MNU + AST group than that in the MNU group. Moreover, the OFF pathway was more efficiently preserved than the ON pathway. (E) The mRNA levels of Caspase-3, Calpain-2, and Bax in the MNU + AST group were significantly down-regulated compared with the MNU group. The mRNA level of Bcl-2 in the MNU + AST group was significantly up-regulated than that in the MNU group. Moreover, the retinal levels of MDA and 8-OHdG were significantly lower in the MNU + AST group compared with the MNU + lutein group. The retinal levels of SOD and Mn-SOD were significantly higher in the MNU + AST group compared with the MNU + lutein group. (ANOVA analysis followed by Bonferroni’s post hoc analysis was performed, *p < .05, ^#^*p* < .01, for differences between groups; *n* = 10).

The spontaneous firing spikes of RGCs were detected by MEA electrodes (Figure 5(A)). The spontaneous firing rate in the MNU group was significantly higher than that in the normal controls (*p* < .01; *n* = 10, Figure 5(B)), suggesting that the RGCs hyperactivity occurred during RD. The spontaneous firing rate in the MNU + AST group increased significantly compared with the normal controls (*p* < .01; *n* = 10) but was significantly lower compared with the MNU group (*p* < .01; *n* = 10). Subsequently, the light induced firing spikes were evoked by light stimulus (Figure 5(C)). The PSTHs and raster plots of individual units were used to classify the RGCs responses (Sagdullaev & McCall, 2005; Stasheff, 2008). Six categories of RGCs were identified according to their responsive characteristics to light stimulus: (a) these RGCs displayed the brisk, transient response to the onset of full field flash (ON-RGCs); (b) these RGCs displayed similar transient response to the offset of full field flash (OFF-RGCs); (c) these RGCs responded transiently to light onset and offset (ON-OFF RGCs); (d) these RGCs displayed sustained responses predominantly to light onset (Sustained ON RGCs); (e) these RGCs displayed sustained response to light on and offset (Sustained ON-OFF RGCs); (f) these RGCs displayed sluggish response to light offset (Delayed OFF RGCs). The number of spikes occurring during light transition were summed and used to compute the response intensity. The ON response was composed of the spikes from transient ON RGCs, sustained ON RGCs, and the ON component of ON-OFF RGCs; The OFF response was composed of the spikes from transient OFF RGCs, delayed OFF RGCs, and the OFF component of ON-OFF RGCs. The total firing rate decreased significantly in the MNU group than that in the normal controls (*p* < .01; *n* = 10; Figure 5(D)). Moreover, the total firing rate was significantly higher in the MNU + AST group than that in the MNU group. A substantial proportion of the light induced ON and OFF response is retained in the MNU + AST group, suggesting that the basic configurations of visual signal pathways are efficiently preserved. On closer inspection, the OFF pathway was more efficiently preserved than the ON pathway. The ON response intensity was 43.4% of that in the normal controls, while the OFF response intensity was 64.1% of that in the normal controls.

The signal-to-noise ratio (SNR = average driven firing rate/spontaneous background firing rate) is valuable to analyze the efficiency of visual signal transmission. Herein, the SNR value in the MNU group was significantly smaller compared with the normal controls. Both the impaired light-induced response and spontaneous hyperactivity collectively contributed to this reduction (Supplementary file). On the other hand, the SNR value in the MNU + AST group was significantly larger than that in the MNU group (*p* < .01; *n* = 10). The SNR value MNU + AST group was at least 21 folds larger than that in the MNU group, suggesting that the circuits in MNU + AST group could transmit visual signals much more efficiently.

### Mechanisms underlying the AST induced protection

The mRNA levels of Caspase-3, Calpain-2 and Bax in the MNU group were not significantly different from those in the MNU + lutein group (*p* > .05; *n* = 10). On the other hand, the mRNA levels of Caspase-3, Calpain-2, and Bax in the MNU + AST group were significantly down-regulated compared with the MNU group (*p* < .01; *n* = 10; Figure 5(E)). The mRNA level of Bcl-2 was significantly up-regulated in the MNU + AST group than that in the MNU group (*p* < .05; *n* = 10). Moreover, the retinal levels of MDA (a stable metabolite of lipid peroxidation) and 8-OHdG (an indicator of oxidative DNA damage) in the MNU group were distinctly higher compared with the normal control group (*p* < .01; *n* = 10). The retinal MDA concentration was 1.65 ± 0.390 nmol/mg in MNU + AST group versus 4.82 ± 0.504 nmol/mg in the MNU + lutein group, and the difference was statically significant (*p* < .01; *n* = 10). The retinal 8-OHdG concentration was 93.66 ± 8.57 μg/mg in MNU + AST group versus 138.82 ± 9.56 μg/mg in the MNU + lutein group (*p* < .01; *n* = 10). The retinal level of SOD, an endogenous antioxidant was 176.887 ± 22.175 U/mg in the MNU + AST group compared to 73.066 ± 18.242 U/mg for the MNU + lutein group (*p* < .01; *n* = 10). The retinal level of Mn-SOD, a mitochondrial protein with ROS scavenging potency, was 38.21 ± 4.63 U/mg in the MNU + AST group compared to 19.61 ± 4.69 U/mg for the MNU + lutein group (*p* < .01; *n* = 10).

## Discussion

AST functions as a protective molecular against a series of pathologic processes in which the oxidative stress has been implicated (Gammone et al., 2015; Régnier et al., 2015; Galasso et al., 2018). In this study, we show that the emulsifier type can affect the basic physicochemical characteristic of the resulting AST nanodispersions. Among these emulsifiers, Polysorbate 20 produced the AST nanodispersions with smaller particle diameters, narrower size distributions, and higher AST contents. Furthermore, the AST nanodispersions alleviate the photoreceptor degeneration and visual function impairments in a RD animal model with rapid progress rate. These preserved photoreceptors functioned well as corroborated by the ERG results and behavior tests.

Nowadays, treatments for RD remain challenging due to the tremendous genetic heterogeneity and obstacles in accurate genetic characterization (Hartong et al., 2006; Souzeau et al., 2018). Any therapeutic strategy dealing with a specific gene defect is only applicable to a small group of RD patients that sharing the mutual gene mutation. Thus, targeting a pathological process which is applicable to all the RD phenotypes may serve as a more promising alternative therapy. Accumulating evidences suggest that oxidative stress contributes to the photoreceptor apoptosis in RD retinas with different etiologic backgrounds (Anarjan & Tan, 2013; Moreno et al., 2018). The delicate structure, complex function, and energetic oxygen metabolism make photoreceptors extremely vulnerable to oxidative insults. Overproduction of ROS in retina and the inability to element excessive ROS have been implicated in the pathophysiology of MNU induced RD (Tsuruma et al., 2012). This is the rationality to build the therapeutic strategy on AST. AST can ameliorate the oxidative damage through multiple mechanisms, including quenching the singlet oxygen, scavenging the ROS, suppressing membrane lipid peroxidation, and bolster the endogenous anti-oxidant system (Liu et al., 2014; Wu et al., 2015; Yeh et al., 2016). Accordingly, AST might act a powerful molecular that is superior to other carotenoids in protecting photoreceptor from oxidative insults. Moreover, polysorbates could act as nonionic emulsifiers to stabilize the AST nanodispersions via electrostatic and steric mechanisms (Anarjan & Tan, 2013). The nano-AST has both hydrophilic and lipophilic properties, which would ensure a 1.5–1.8 times higher plasmatic AST contents than traditional AST emulsion (Harada et al., 2017). These advantages would collectively contribute to the potency of AST nanodispersions.

Apoptosis is the ultimate fate of photoreceptors in various RD retinas. Photoreceptor apoptosis would reduce the oxygen consumption and exacerbate the oxidative stress in retinal tissue. On the other hand, oxidative stress in turn activates the apoptotic cascade, and accelerates the destruction of photoreceptors (Campochiaro & Mir, 2018). Generally, TUNEL assay acts a reliable method to identify the apoptotic cells and quantify the apoptotic levels in retinas (Nagar et al., 2017). We find that AST nanodispersions can reduce significantly the mRNA level of Caspase-3, which acts as a classic mediator in photoreceptor apoptosis. AST nanodispersions also enhance the Bcl-2 expressions and adjust the Bcl/Bax ratio toward a net ‘anti-apoptotic’ effect. Consistent with our findings, a previous study has demonstrated that AST treatment attenuates RGCs apoptosis in a mice model of type-2 diabetes (Dong et al., 2013). AST treatment can also inhibit H_2_O_2_-mediated neural progenitor cell apoptosis through the modulation of p38/MEK signaling pathway (Kim et al., 2009). An independent study also shows that AST treatment suppresses neuron apoptosis in a rat subarachnoid hemorrhage model through modulating the Akt/Bad pathway (Zhang et al., 2014). Further studies are necessary to characterize the exact pathways in which AST blocks the activation of apoptotic signaling.

As a natural compound, AST is relatively safe and appropriate for chronic application. Currently, several types of AST are commercially available in the forms of nutritional supplements and food additives (Ambati et al., 2014; Régnier et al., 2015). It is well-tolerated in various laboratory experiments and clinical trials without obvious side effects (Fassett & Coombes, 2011). In view of these advantages and safety profiles, AST nanodispersions might be developed into a promising therapeutic candidate for clinical use. Given the variability in the genetic background of RD, a large-scale study would be necessary to characterize the optimal dosages, administration routes, and therapeutic time window of AST therapy. Moreover, potential adverse effects of the high-dose AST for RD should be evaluated by a randomized, controlled, double-masked clinical trial.

MEA technology can detects simultaneously the electrophysiology activity of regional retinal neurons, and acts as an objective tool to evaluate therapeutic efficiency (Zeck, 2018). Photoreceptors of the MNU administered mice show comparative sensitivities to AST therapy: the photoreceptors in peripheral retina are more efficiently preserved than those in the central and mid-peripheral retina. On closer inspection, the electrophysiological function of the inner retinal circuits is examined. The spontaneous RGCs hyperactivity occurs during the MNU induced RD. The spontaneous RGCs hyperactivity is detrimental since it would add undesirable noise to the visual signal transmission within retinal circuits (Pu et al., 2006; Sekirnjak et al., 2011; Stasheff et al., 2011). Together with the impaired light induced response, these disturbances would attenuate the SNR in the MNU administered mice (Barrett et al., 2015). Generally, RGCs collect field potentials from presynaptic inputs and convert them into firing spikes. The success of any therapeutic strategy ultimately depends on the functional recovery of RGC outputs (Barrett et al., 2015; Strettoi, 2015). AST therapy can alter the electrophysiological function of RGCs and restrain the spontaneous hyperactivity in MNU administered mice. Moreover, a substantial proportion of the light induced response is retained in the AST treated mice, indicating that the basic configurations of visual signal pathways are efficiently preserved (Puchalla et al., 2005; Marc et al., 2007). Intriguingly, the visual pathway reorganization is found in the AST treated mice. The intrinsic balanced between ON and OFF pathways has been disturbed, and the OFF signal pathway would dominate the visual signal transmission in visual circuits. The basement underlying this signal pathway reorganization should be attributed to the plasticity of retinal circuits (Lin et al., 2009; Bisti, 2010). Generally, the hyperpolarization of OFF pathway would be beneficial for retinal neurons to detect small changes in light intensity at low illumination level (Zhao et al., 2010). In retinal circuits, the glycinergic amacrine cells are critically involved in the crossover inhibition between ON and OFF pathways (Hsueh et al., 2008). It is possible that AST treatment adjusts the OFF signal pathway via the glycinergic inputs from amacrine cells. Further studies are necessary to characterize the exact mechanism underlying this modulation.

In conclusion, AST nanodispersions can alleviate the photoreceptor loss and visual impairments in the MNU administered mice. AST nanodispersions afford these protective effects by modulating apoptosis and alleviating oxidative stress. These findings indicate that AST nanodispersions may act as a therapeutic strategy to ensure the successful rescue of visual function. Further studies are necessary to provide a framework to understand the therapeutic potentials of AST.

## Supplementary Material

Supplemental Material
