# Supplementary figures and images for: Optimized nonionic emulsifier for the efficient delivery of astaxanthin nanodispersions to retina: in vivo and ex vivo evaluations
Source: Drug Deliv. 2019 Nov 21;26(1):1222–34. doi: 10.1080/10717544.2019.1682718 (PMC6882443; doi:10.1080/10717544.2019.1682718)

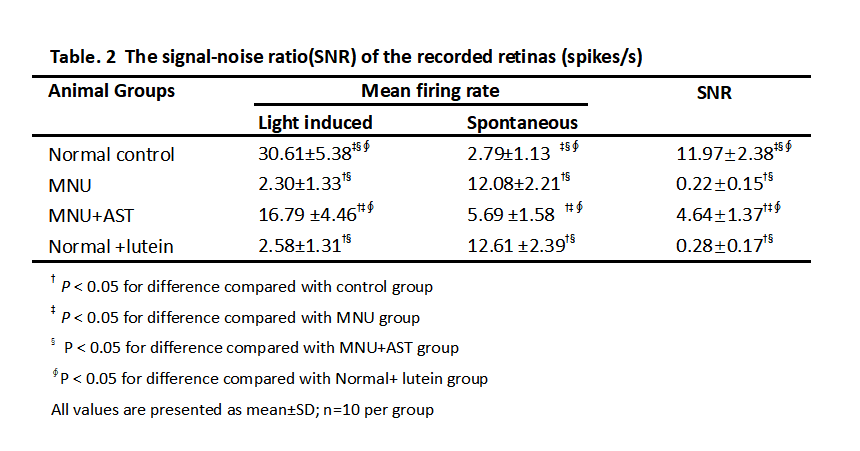

Supplement: Supplemental Material [file IDRD_A_1682718_SM9714.png]
